# Unlocking the longevity code with stress

**DOI:** 10.7554/eLife.109050

**Published:** 2025-10-06

**Authors:** Loren Cocciolone, Valeria Uvarova, Patricija van Oosten-Hawle

**Affiliations:** 1 Department of Biological Sciences, University of North Carolina at Charlotte Charlotte United States

**Keywords:** aging, hypoxic stress response, HIF-1, TYRA-3, FMO-2, *C. elegans*

## Abstract

Manipulating the neuronal pathway responsible for the hypoxic stress response in the worm *C. elegans* leads to an increase in lifespan.

**Related research article** Kitto ES, Huang S, Bhandari M, Tian C, Cox RL, Beydoun S, Wang E, Shave D, Miller HA, Easow SA, Henry E, Schaller ML, Leiser SF. 2025. The hypoxic response extends lifespan through a bioaminergic and peptidergic neural circuit. *eLife* **14**:RP107651. doi: 10.7554/eLife.107651.

We all know the basics of healthy aging: eat well, do exercise and get enough sleep. However, research has revealed that aging is not simply a passive and inevitable decline. Rather, it is a process that can be delayed by targeting certain signaling pathways, such as the pathways that help the body respond and adapt to various forms of stress that might otherwise cause harm. In particular, it has been shown that caloric restriction (that is, reducing food intake without causing malnutrition) can delay aging, as can exposures to heat or cold. The basic idea of this approach, which is called hormesis, is that small amounts of stress can be like ‘training exercises’ that make the body more resilient and, ultimately, extend lifespan (Calabrese, 2014).

In multicellular organisms, stress responses are coordinated within the individual cells that perceive the stress, and also across tissues. The nervous system plays a central role here, sensing the stress and using chemical messengers to orchestrate protective responses throughout the body. Within the context of aging, neuronal signals regulate how cells respond to a shortage of food, heat stress and even misfolded proteins (Miller et al., 2022; O’Brien et al., 2018; Özbey et al., 2020; Prahlad et al., 2008). Because of these discoveries, there has been growing interest in targeting neural circuits to improve health and longevity.

One stress response pathway that has been underexplored within the context of longevity is the response to low levels of oxygen, also known as the hypoxic response. When oxygen levels drop, a protein called HIF-1 is activated and switches on cellular survival programs leading to metabolic rewiring (Semenza, 2012a). This is a highly conserved response that has been observed across a wide range of species, highlighting its importance for survival (Kaelin and Ratcliffe, 2008). It has been shown that stabilizing HIF-1, either through genetic modification or by hypoxia itself, can extend lifespan and improve health (Leiser et al., 2015).

If this sounds too good to be true, it probably is. Here is the caveat: if HIF-1 is activated for too long, the same mechanism that protects against hypoxic stress can have detrimental effects, including tumor progression and cardiovascular disease (Semenza, 2012b). Thus, the key challenge is to understand and harness the precise neuronal signals that allow hypoxia to confer benefits, without causing disease.

Now, in eLife, Scott Leiser (University of Michigan) and colleagues – including Elizabeth Kitto (Michigan) and Shijiao Huang (Kansas State University) as joint first authors – report that they have mapped the neuronal pathway responsible for the hypoxic stress response in the worm *C. elegans,* and then manipulated specific genes and neurons to increase lifespan in this species (Kitto et al., 2025). They discovered that signals originate in serotonin-producing neurons in the head of the worm, and travel through a chain of neurons using messenger chemicals such as GABA, tyramine (the worm’s version of adrenaline) and neuropeptides. All these signals eventually converge on the intestine, where they bind to a tyramine receptor called TYRA-3, and activate an enzyme called FMO-2, which contributes to lifespan extension (Figure 1). Kitto et al. show how stress perceived and sensed in one part of the body (hypoxia sensed by neurons in the head) can send protective signals to distant cells (in the intestine) to boost the resilience of the whole organism.

**Figure 1.**
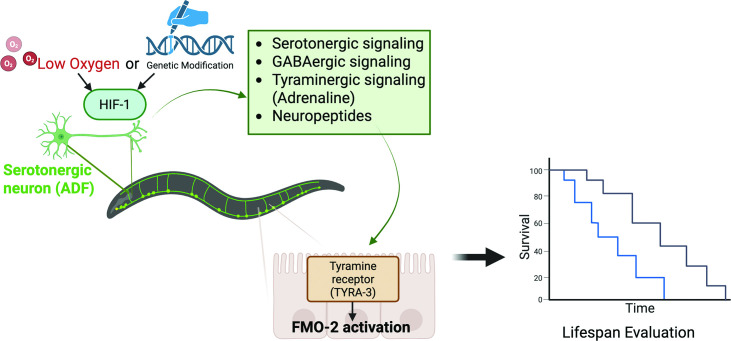
Hypoxia-mediated neuronal longevity signaling. Low-oxygen stress (hypoxia) or genetic modification of a protein called HIF-1 in neurons induces a neuronal signaling cascade involving a number of different chemical messengers (serotonin, GABA, tyramine and various neuropeptides). These signaling pathways converge on intestinal cells, where the messengers bind to the tyramine receptor TYRA-3, and switch on the enzyme FMO-2, which contributes to lifespan extension. The blue line in the graph shows survival rate (vertical axis) as a function of time in the absence of stress; the black line shows how stress increases the survival rate, and hence the lifespan. Created with BioRender.

Overall, these results provide a blueprint for how stress responses could be harnessed to improve health and longevity in other species in the future. The next step will be to investigate potential therapeutic targets within the hypoxic stress response pathway in mammals for increasing lifespan while limiting negative impacts.

One potential target is an interneuron called RIS that has been found to extend lifespan when its activity is increased (Busack and Bringmann, 2023). However, perhaps the most promising target is the HIF-1 protein itself: Kitto et al. show that stabilizing this protein in ADF neurons leads to a 26% increase in the lifespan of *C. elegans*. Of course, caution is needed: an approach that helps worms to live longer might cause unwelcome side effects in humans. However, now that the hypoxic stress response pathway in *C. elegans* has been mapped, we have targets that can be explored in more detail in mammals.

In the end, the work of Kitto et al. reminds us that stress is not always bad. In small doses it can activate otherwise ‘hidden’ cellular programs of resilience. The challenge in the future will be to learn how to switch those programs on, in just the right amount and at the right time, to help us live longer, healthier lives.
